# Unprecedented Fluorescent Dinuclear Co^II^ and Zn^II^ Coordination Compounds with a Symmetric Bis(salamo)-Like Tetraoxime

**DOI:** 10.3390/molecules23051141

**Published:** 2018-05-10

**Authors:** Lin-Wei Zhang, Ling-Zhi Liu, Fei Wang, Wen-Kui Dong

**Affiliations:** School of Chemical and Biological Engineering, Lanzhou Jiaotong University, Lanzhou, 730070, China; zhanglinwei@mail.lzjtu.cn (L.-W.Z.); llz1009663202@126.com (L.-Z.L.); wangfei3986@163.com (F.W.)

**Keywords:** bis(salamo)-like tetraoxime, coordination compound, synthesis, structure, fluorescence property

## Abstract

Two unprecedented homometallic Co^II^ and Zn^II^ coordination compounds, [M_2_(L)(OCH_3_)][M_2_(L)(OAc)] (M^II^ = Co^II^ (**1**) and Zn^II^ (**2**)), with a novel symmetric bis(salamo)-like tetraoxime ligand H_3_L were synthesized and characterized by elemental analyses, infrafred (IR), ultraviolet–visible spectroscopy (UV-Vis), fluorescent spectra and single-crystal X-ray diffraction analyses. The unit cell of the two coordination compounds contains two crystallographically and chemically independent dinuclear coordination compounds. In the two coordination compounds, three metal ions are five-coordinated, formed two square pyramidal and a trigonal bipyramidal geometries, and the other metal ion is a hexacoordinate octahedral configuration. In addition, the coordination compound **1** forms a 3D supramolecular structure, and the coordination compound **2** forms a 0D dimer structure by the inter-molecular hydrogen bond interactions. Meanwhile, the fluorescence spectra of the coordination compounds **1** and **2** were also measured and discussed.

## 1. Introduction

As we know, salen-like ligands play an important role in the field of inorganic chemistry [1,2,3,4,5,6,7,8]. They are synthesized by the interaction of diamines with salicylaldehyde or its derivatives, and can coordinate to transition metal ions in a tetradentate fashion to obtain mono- or polynuclear metal coordination compounds [9,10,11,12]. These coordination compounds have been extensively investigated as nonlinear optical materials [13], catalysts [14], biological systems [15], magnetic materials [16], supramolecular buildings [17,18,19,20,21,22,23,24,25,26], and so on. In order to improve the structure of salen-like ligands and strengthen the coordination ability, in recent years, our research has mainly concentrated on the syntheses of salamo-like ligands and their metal coordination compounds. A new study has shown that it is at least 10^4^ times more stable against the metathesis reaction in H_2_O/MeCN (5:95) at 40 °C than salen-like coordination compounds due to the unique structure of salamo-like coordination compounds [27]. In our previous studies on salamo-type metal complexes, we exchanged salicylaldehyde for its derivatives to obtain some new salamo-like transition metal coordination compounds with different structures [28,29,30,31,32]. The structural motifs of these coordination compounds may be affected by the performance of the ligands, the property of the central atoms, solvent effect, anion effect and so forth [33,34,35,36,37,38,39,40,41,42]. In addition, some practical photophysical properties of transition metal coordination compounds with salamo-like bisoxime ligands have been reported in succession [43,44,45,46,47,48,49,50]. The N_2_O_2_ tetradentate motif can coordinate easily with transition metal ions. Therefore, salamo-like ligands can form mono-, di- or trinuclear metal coordination compounds with transition metal ions. Meanwhile, some Co^II^ and Zn^II^ salamo-like coordination compounds have been reported earlier [51,52,53,54,55,56,57].

The aim of the present work is the structural characterization of the homometallic coordination compounds **1** and **2** based on a symmetric bis(salamo)-like tetraoxime ligand. Herein, the ligand H_3_L and its corresponding dinuclear coordination compounds **1** and **2** were prepared successfully. Interestingly, the obtained 2:1 (metal-to-ligand stoichiometry) type coordination compounds are unusual in the previously reported bis(salamo)-type metal coordination compounds, in which most of them possess 3:1 (metal-to-ligand stoichiometry) type of structures [43,57]. Furthermore, the supramolecular features and luminescent spectra of the coordination compounds **1** and **2** are discussed.

## 2. Results and Discussion

### 2.1. Crystal Structures of the Coordination Compounds **1** and **2**

**Figure 1:**
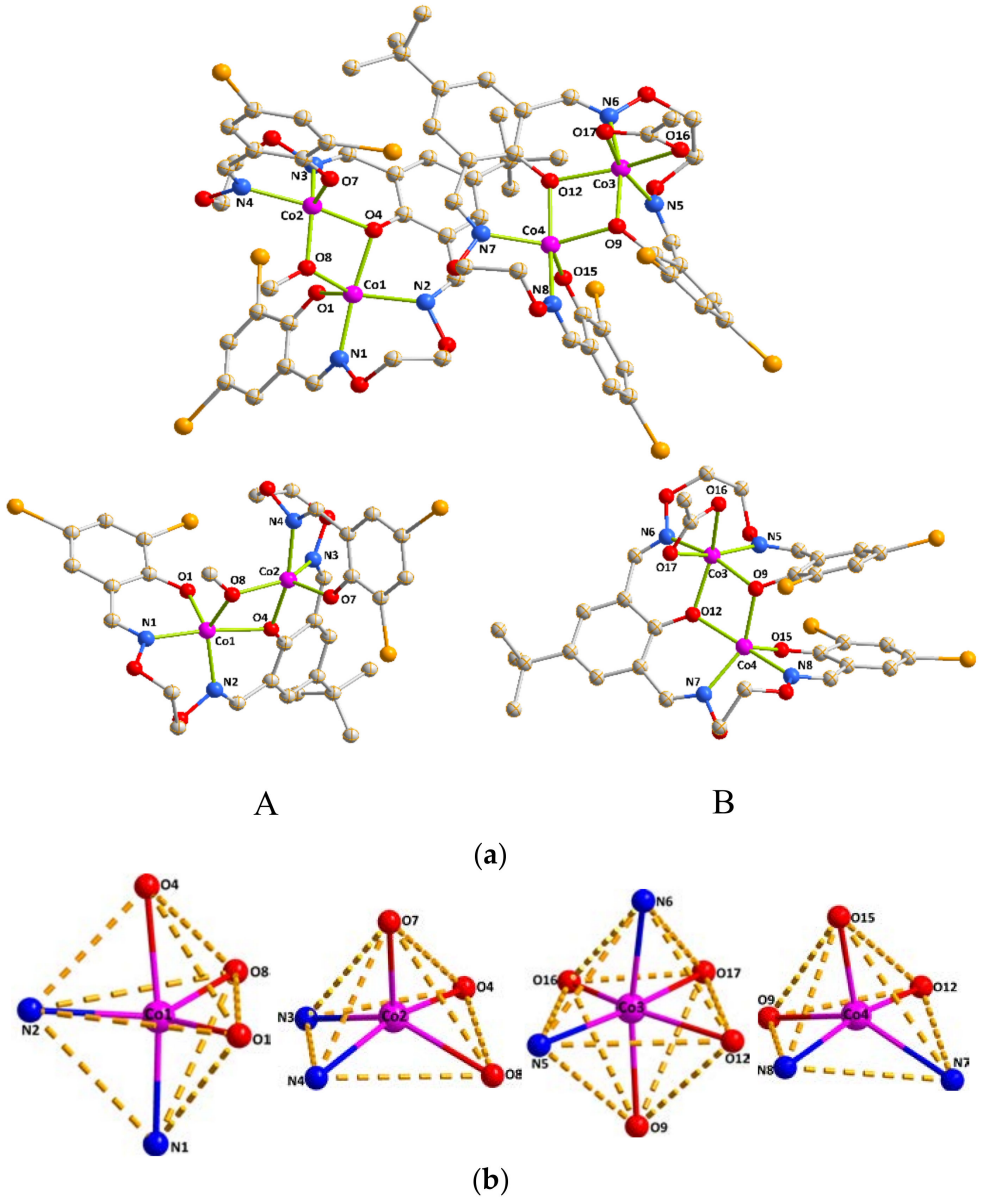
(**a**) Molecule structure and atom numberings of the coordination compound **1** (hydrogen atoms are omitted for clarity). (**b**) Coordination polyhedrons for Co^II^ atoms of the coordination compound **1**.

**Figure 2:**
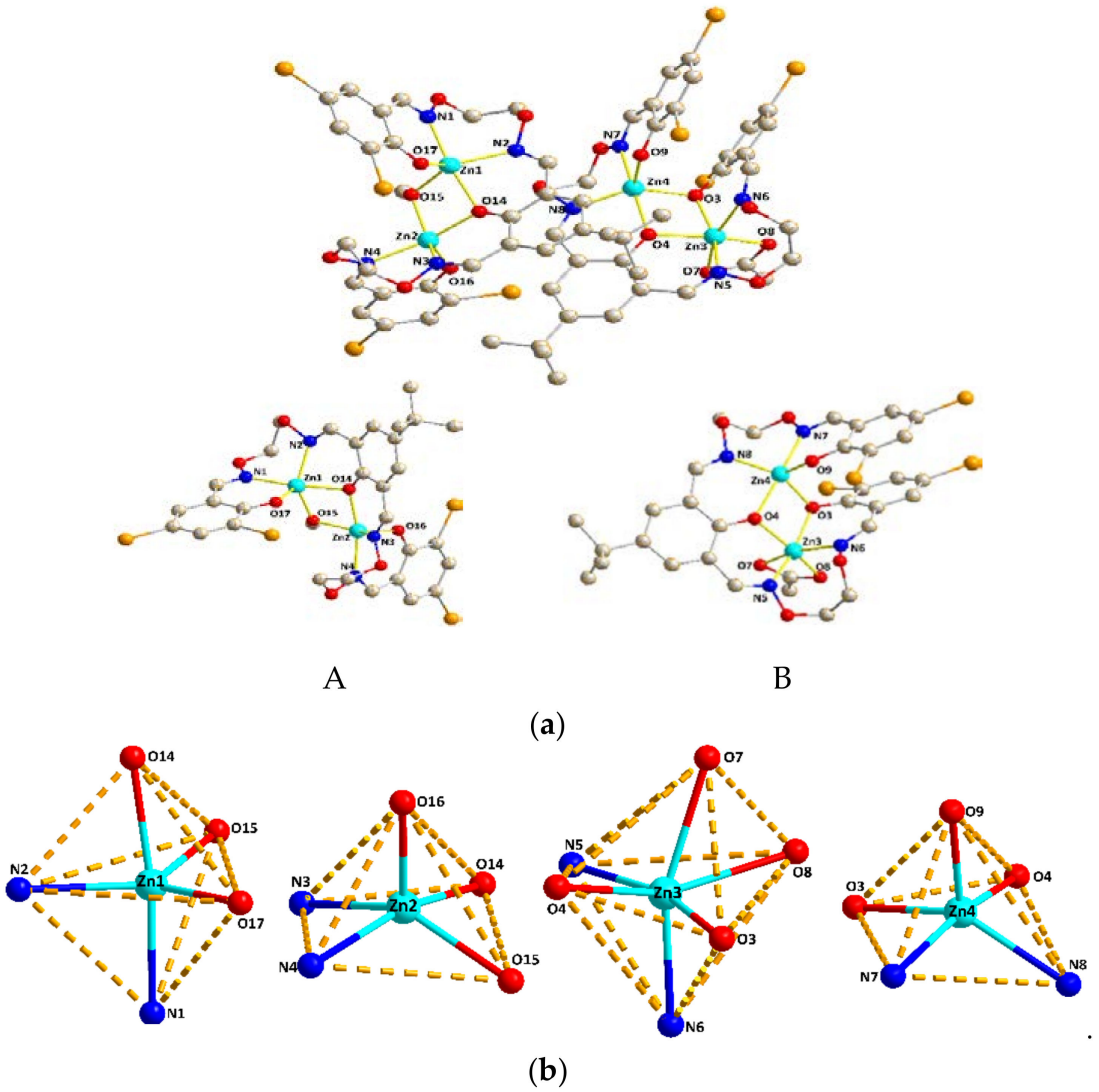
(**a**) Molecule structure and atom numberings of the coordination compound **2** (hydrogen atoms are omitted for clarity). (**b**) Coordination polyhedrons for Zn^II^ atoms of the coordination compound **2**.

X-ray crystallographic analyses reveal that the structure of the coordination compound **2** is similar to that of the coordination compound **1**. The coordination compounds **1** and **2** form novel dinuclear structures, which are different from the common trinuclear structures of bis(salamo)-like metal coordination compounds reported earlier [58,59,60,61,62]. The crystal structures of the coordination compounds **1** and **2** and the coordination polyhedrons of the M^II^ atoms are shown in Figure 1 and Figure 2. Selected bond lengths and angles are listed in Table 1 and Table S1.

The coordination compounds **1** and **2** crystallize in the triclinic crystal system, space group *P*-1, and the unit cell of the two coordination compounds contains two crystallographically and chemically independent dinuclear coordination compounds (A and B molecules) (As shown in Figure 1 and Figure 2). In the two coordination compounds, A molecule consisting of two M^II^ atoms, one heptadentate (L)^3−^ unit and one *μ*_2_-bridged methoxyl group, and B molecule is composed of two M^II^ atoms, one heptadentate (L)^3−^ unit and one chelating acetate ion. In molecules A and B, the purpose of the acetate ion and methoxyl group is to compensate for the charge and make the whole molecule neutral. The obtained 2:1 (metal-to-ligand stoichiometry) type dinuclear coordination compounds are unprecedented in the reported bis(salamo)-like metal coordination compounds, which always possess 3:1 (metal-to-ligand stoichiometry) type of structures [43,57].

In each of the A molecules, all of the M^II^ atoms are located in the N_2_O_2_ coordination spheres of the salamo-type ligand (L)^3−^ unit, the *μ*_2_-bridged methoxyl groups bridge two M^II^ atoms in a familiar M‒O‒M fashion (Figure 1a and Figure 2a). Meanwhile, two M^II^ atoms of the coordination compounds **1** and **2** are pentacoordinated and adopt distorted trigonal bipyramidal (Co1 and Zn1) and square pyramidal (Co2 and Zn2) geometries (Figure 1b and Figure 2b), which were deduced by calculating the values of *τ*_Co1_ = 0.62, *τ*_Co2_ = 0.41, *τ*_Zn1_ = 0.63 and *τ*_Zn2_ = 0.41, respectively [63]. From the calculation results, we can see that the *τ* values of Co1 and Zn1 are greater than 0.5, forming trigonal bipyramidal configurations, and the *τ* values of Co2 and Zn2 are less than 0.5, forming square pyramidal configurations. The structures of the B molecules are different from those of the A molecules, the Co4 and Zn4 atoms of the coordination compounds **1** and **2** are pentacoordinated and adopt distorted square pyramidal geometries, which were deduced by calculating the values of *τ*_Co4_ = 0.49 and *τ*_Zn4_ = 0.48, respectively. The Co4 and Zn4 atoms are located in the N_2_O_2_ coordination spheres of the salamo-type ligand (L)^3−^ unit, and coordinate to one phenoxo oxygen (O9) atom, respectively. The Co3 and Zn3 atoms coordinate to N_2_O_2_ atoms of the deprotonated ligand (L)^3−^ units as well as two oxygen atoms from one chelating acetate ion, and have a hexacoordinated environment and adopt distorted octahedral coordination geometries (By means of continuous shape measures (CShM), when the value of CShM is the smallest, the ideal structure is the octahedron configuration, CShM = 3.03270 and 3.72885 for Co3 and Zn3 atoms) [64].

**Figure 3:**
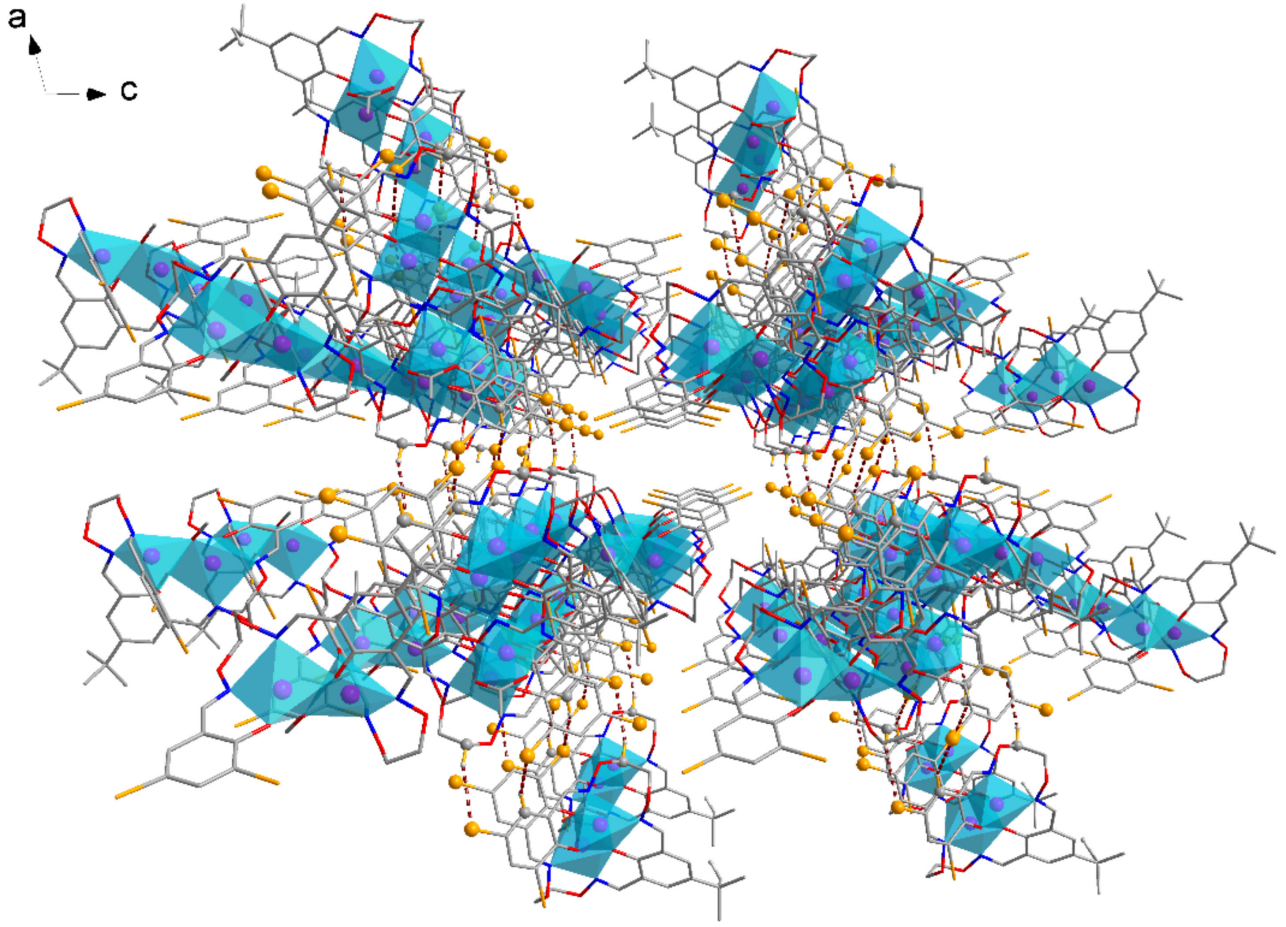
The 3D supramolecular structure of the coordination compound **1** with inter-molecular hydrogen bondings (hydrogen atoms, except those forming hydrogen bonds, are omitted for clarity).

**Figure 4:**
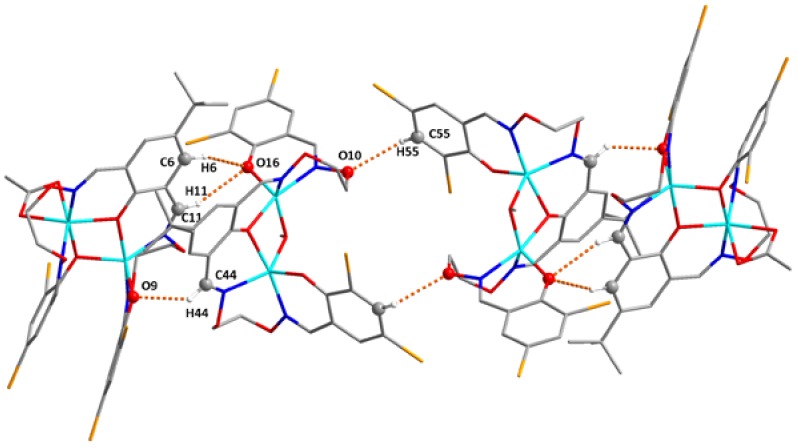
The 0D dimer structure of the coordination compound **2** with inter-molecular hydrogen bondings (hydrogen atoms, except those forming hydrogen bonds, are omitted for clarity).

The supramolecular structures of the coordination compounds **1** and **2** are very different from each other. In the crystal structure of the coordination compound **1**, there are eight significant intermolecular hydrogen bonds (C9‒H9A···O13, C10‒H10···O15, C40‒H40A···Br8, C61‒H61A···Br2, C36‒H36···Br7, C8‒H8A···Br8, C39‒H39B···Br6 and C43‒H43···O7) and one intramolecular hydrogen bond (C49‒H49A···O16). The units are interlinked by the intermolecular C9‒H9A···O13, C10‒H10···O15, C40‒H40A···Br8, C61‒H61A···Br2 and C43‒H43···O7 hydrogen bonds into a 2D layered supramolecular structure, which are further assembled into an infinite 3D network structure with the help of intermolecular C36‒H36···Br7, C8‒H8A···Br8 and C39‒H39B···Br6 hydrogen bond interactions (Figure 3). For the coordination compound **2**, there is a pairs of intermolecular hydrogen bond (C55‒H55···O10). The oxygen (O10) atom of the (L)^3−^ unit is hydrogen bonded to the C55–H55 group of another coordination compound **2** molecule, linking a 0D dimer structure (Figure 4). In addition, A and B molecules are connected steadily by intermolecular C‒H···O hydrogen bond interactions. Putative hydrogen bond interactions for the coordination compounds **1** and **2** are shown in Table 2.

### 2.2. IR Spectra

IR spectra of H_3_L and its corresponding coordination compounds **1** and **2** exhibit various bands in the region of 400–4000 cm^−1^. Main IR bands/cm^−1^ for the ligand H_3_L and its coordination compounds **1** and **2** are presented in Table 3.

The free ligand H_3_L shows a characteristic C=N stretching band at 1611 cm^−1^, while the C=N stretching bands of the coordination compounds **1** and **2** appear at 1619 and 1621 cm^−1^, respectively [65]. For the ligand H_3_L, the Ar‒O stretching band appears at 1265 cm^−1^, which is observed at 1258 and 1261 cm^−1^ for the coordination compounds **1** and **2**. The characteristic C=N and Ar–O stretching frequencies are shifted to lower frequencies, indicating that the M–N and M–O bonds are formed [66]. For the coordination compound **1**, the *ν*(Co–O) and *ν*(Co–N) frequencies are observed at 447 and 512 cm^−1^, respectively [67]. Meanwhile, the *ν*(Zn–O) and *ν*(Zn–N) bonds at 453 and 519 cm^−1^ for the coordination compound **2**. As pointed out by *Percy* and *Thornton* [68], the M‒O and M‒N frequency assignments are at times difficult.

### 2.3. Ultraviolet–Visible Spectroscopy (UV-Vis) Spectra

**Figure 5:**
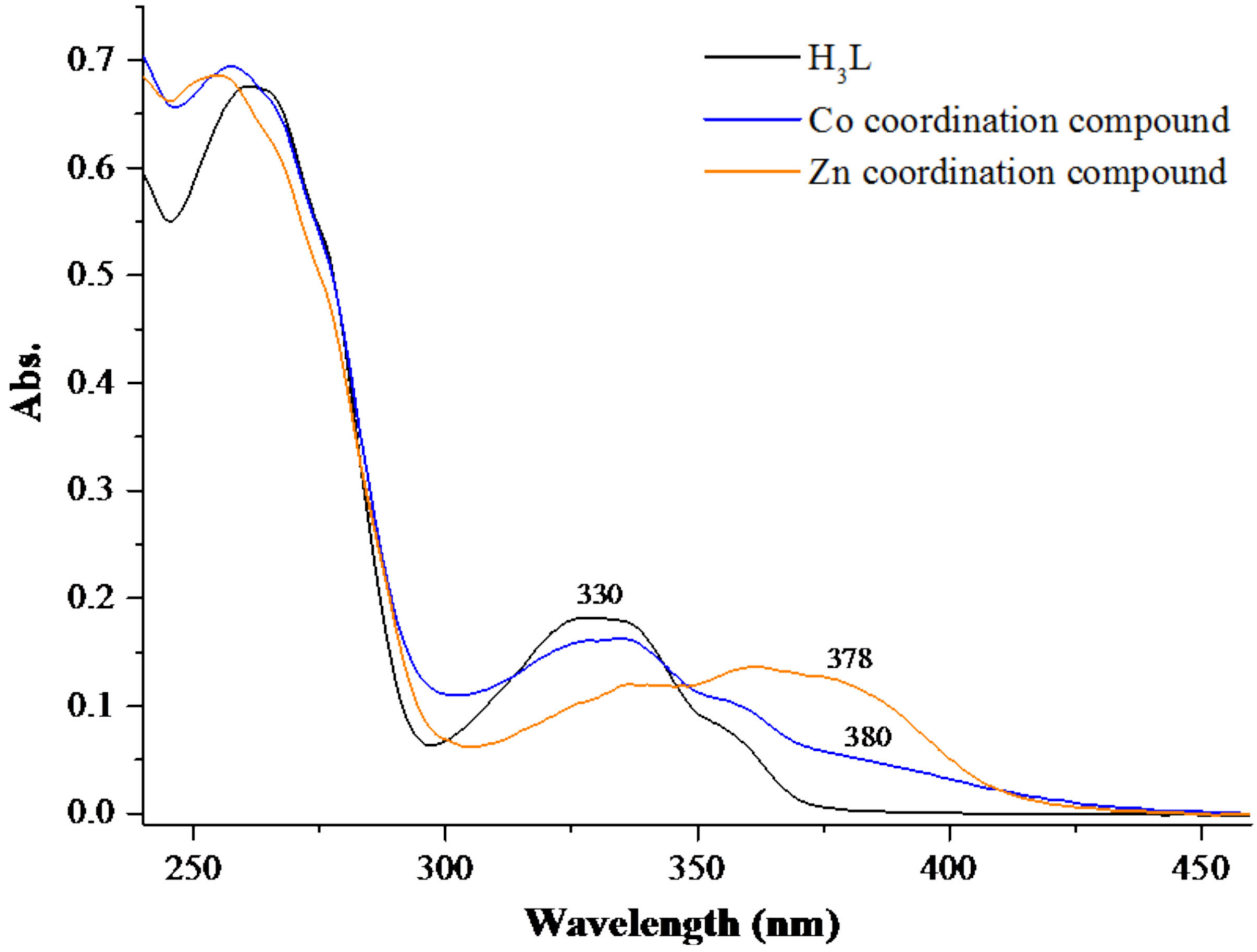
Ultraviolet–visible spectroscopy UV-Vis absorption spectra of H_3_L and its coordination compounds **1** and **2** in MeOH (1 × 10^−5^ M).

The UV-Vis absorption spectra of H_3_L and its coordination compounds **1** and **2** were determined in 1 × 10^−5^ mol·L^−1^ MeOH solution, as shown in Figure 5. It can be seen that the absorption peaks of the coordination compounds **1** and **2** are obviously different from those of the H_3_L upon coordination. The electronic absorption spectrum of H_3_L consists of one relatively intense peak centered at 330 nm, assigned to the π–π* transition of the oxime groups [69,70]. Compared with the absorption peak of the free ligand H_3_L, the corresponding absorption peaks of the coordination compounds **1** and **2** appear at 380 and 378 nm, which are bathochromically shifted by 50 and 48 nm, respectively, indicating the coordination of the Co^II^ and Zn^II^ ions with the ligand H_3_L.

**Figure 6:**
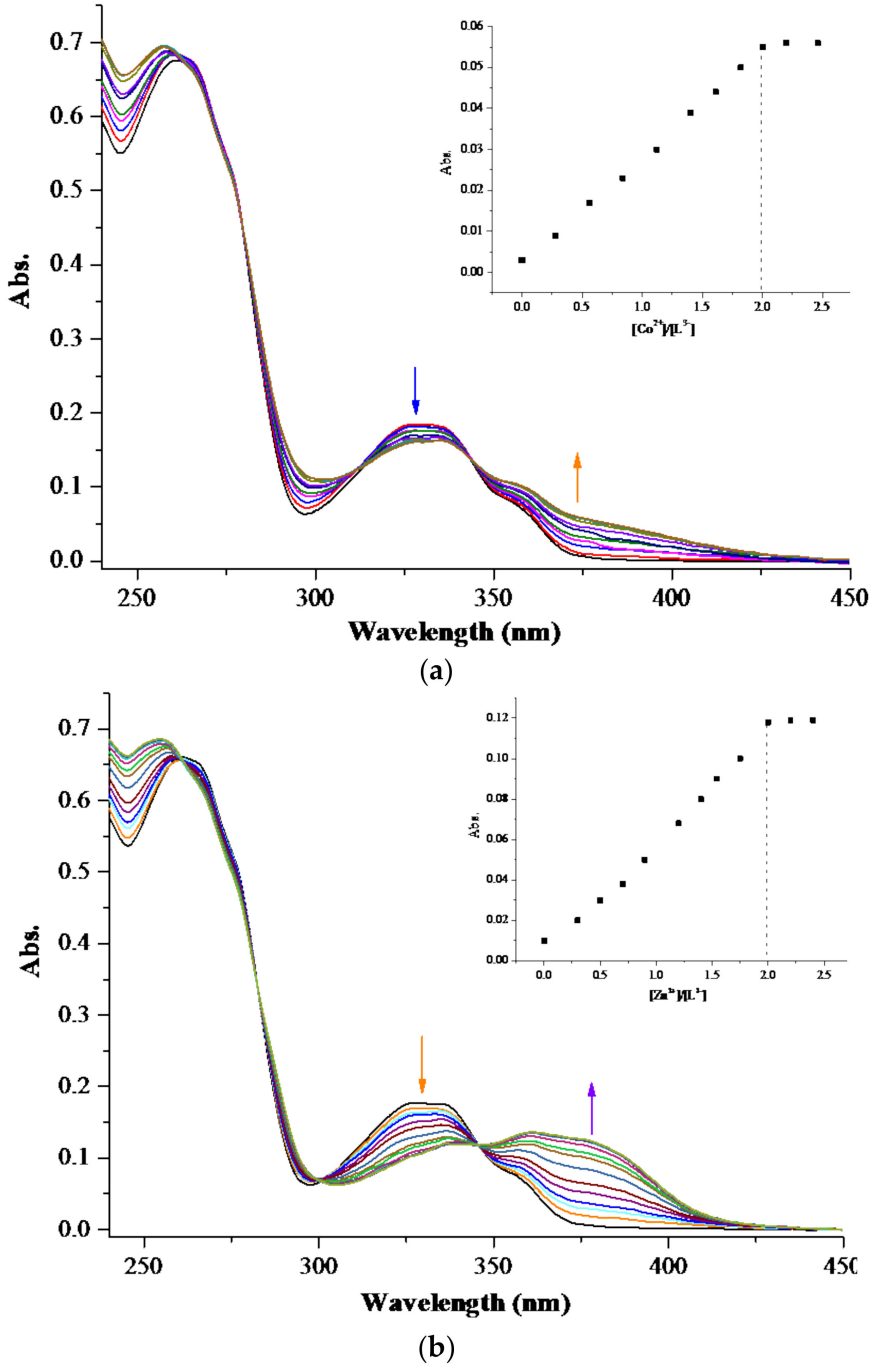
UV-Vis spectral changes of the coordination compounds **1** (**a**) and **2** (**b**) upon addition of different amounts of Co^II^ and Zn^II^ ions (MeOH (1 × 10^−5^ M)).

In the UV-Vis titration experiment of the coordination compound **1**, with the increasing concentration of Co^2+^, the absorbance of the solution at 380 nm enhanced, and at 330 nm reduced. The absorption peak reached the highest value after Co^2+^ was added up to 2 equiv. The spectroscopic titration indicates that the ratio of the replacement reaction was 2:1 (Co^2+^: L^3−^). Similar changes also appear in the coordination compound **2**, obtaining the same conclusion (Figure 6).

### 2.4. Fluorescence Spectra

**Figure 7:**
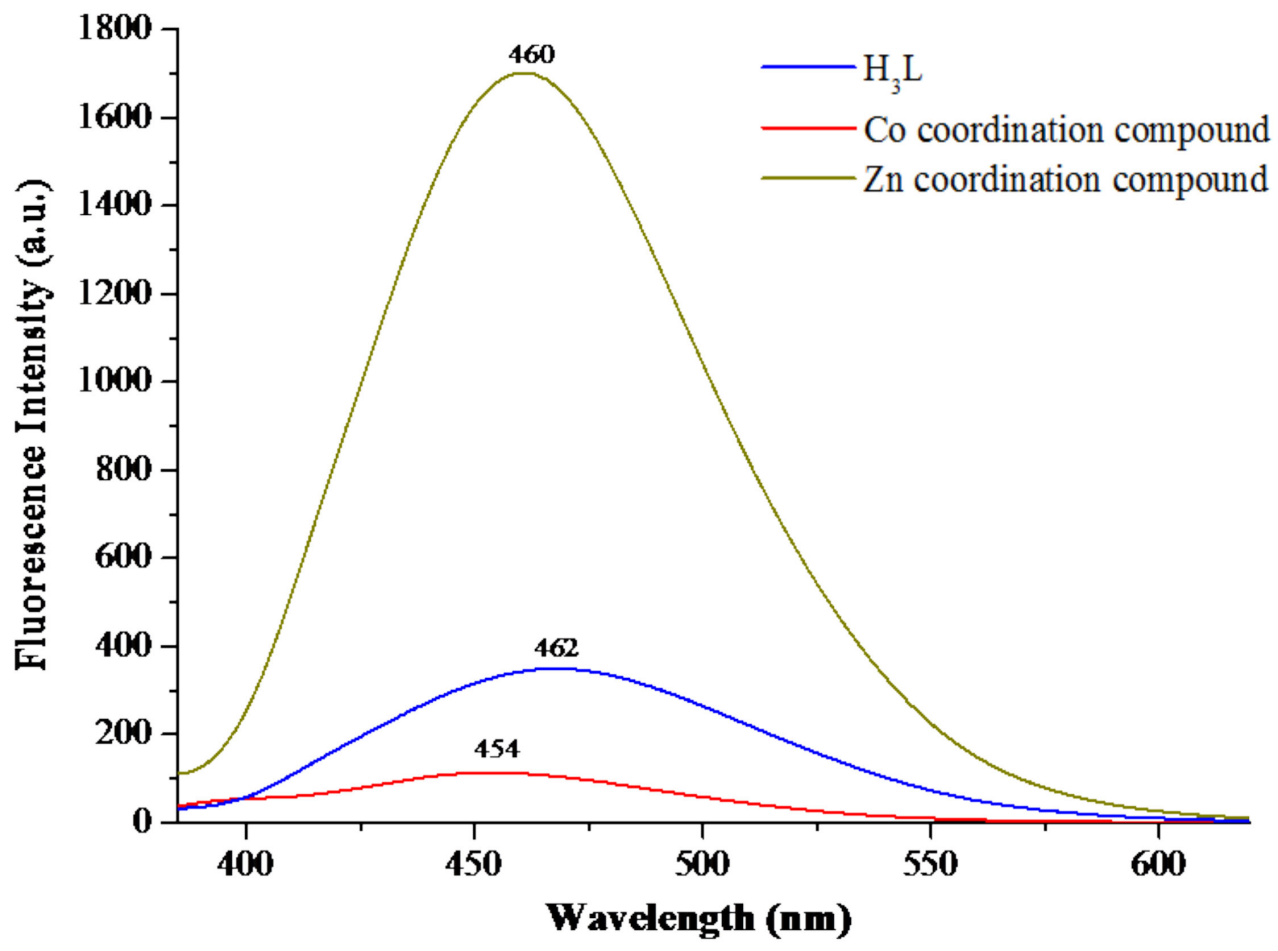
Emission spectra of H_3_L (c = 1 × 10^−5^ M, *λ*_ex_ = 370 nm) and its coordination compounds **1** and **2** in dilute MeOH solutions at room temperature.

The fluorescence spectra of H_3_L and its corresponding coordination compounds **1** and **2** were investigated at room temperature and are shown in Figure 7. The free ligand H_3_L exhibits a relatively strong emission peak at ca. 462 nm upon excitation at 370 nm, and it should be assigned to the intraligand π–π^*^ transition. The coordination compound **1** shows lower photoluminescence with maximum emission at ca. 454 nm. Compared with the ligand H_3_L, emission intensity of the coordination compound **1** reduces obviously, indicating that the Co^II^ ions have a quality of fluorescent quenching, which makes the conjugated system larger and also indicates it may be a purple device. On the other hand, the coordination compound **2** shows an obvious fluorescence enhancement at ca. 460 nm. The intense peak is likely due to the coordination of H_3_L with the Zn^II^ ions, which breaks the intramolecular hydrogen-bonding interactions of H_3_L and increases the coplanarity of the conjugated system.

**Figure 8:**
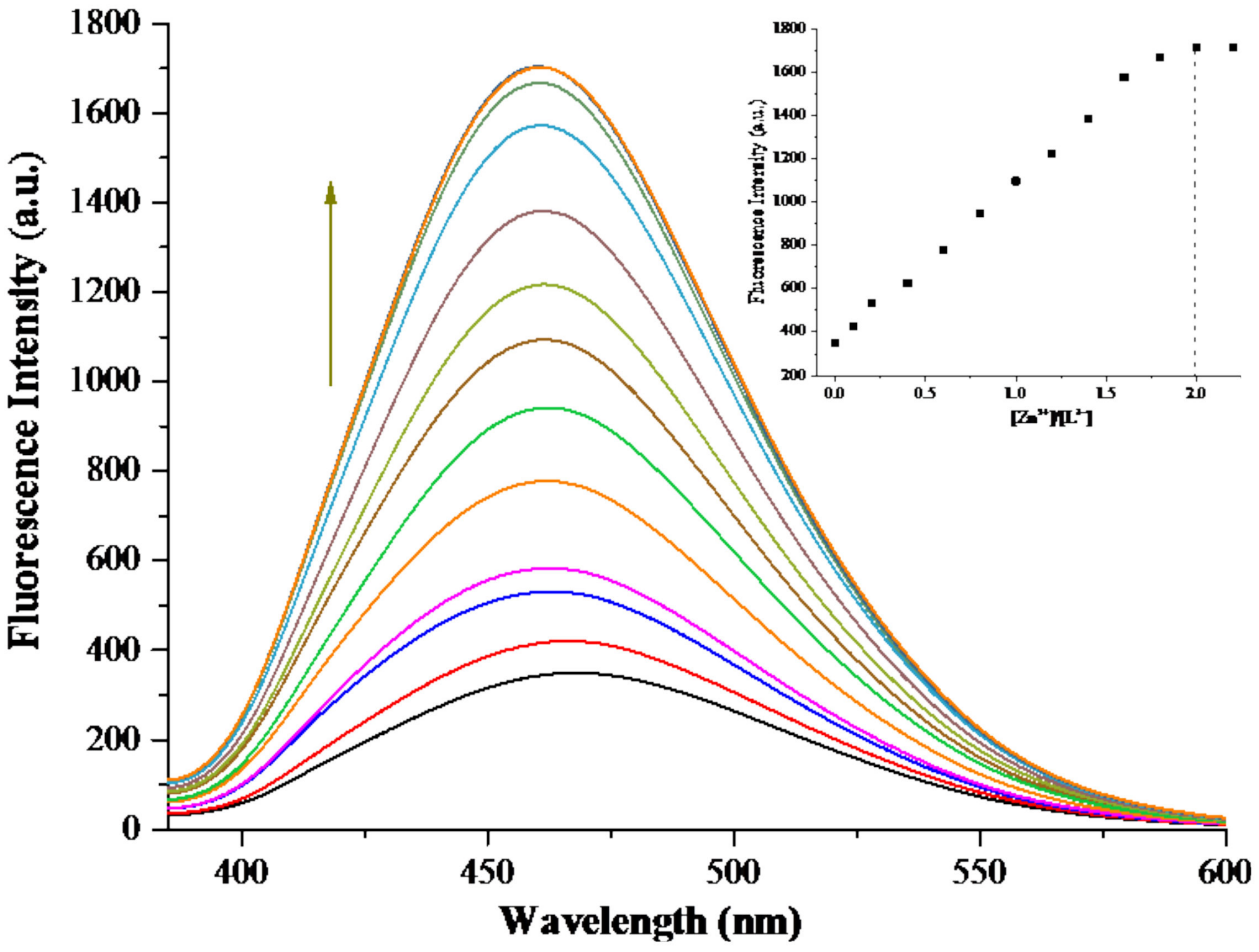
Fluorescence spectrum changes of H_3_L (c = 1 × 10^−5^ M, *λ*_ex_ = 370 nm) solution upon addition of different amounts of Zn^II^ ions.

In addition, the fluorescence titration experiment of the coordination compound **2** is shown in Figure 8. The fluorescence intensity of the solution hardly changed after the Zn^II^ ions were added up to 2 equiv, which shows the same conclusion compared with the UV-Vis titration experiment. Meanwhile, coordination of the Zn^II^ ions evidently increases the fluorescence intensity of the ligand H_3_L.

## 3. Experimental

### 3.1. Materials and Physical Measurements

All chemicals were of analytical reagent grade and were used without further purification. C, H, and N analyses were obtained using a GmbH VarioEL V3.00 automatic elemental analysis instrument (Berlin, Germany). Elemental analyses for Co and Zn were detected by an IRIS ER/S·WP-1 ICP atomic emission spectrometer (Berlin, Germany). Melting points were measured via a microscopic melting point apparatus (Beijing Taike Instrument Limited Company, Beijing, China). ^1^H-NMR spectra were determined by German Bruker AVANCE DRX-400 spectroscopy (Bruker AVANCE, Billerica, MA, USA). Infrared (IR) spectra were recorded with a VERTEX-70 FT-IR spectrophotometer, with samples prepared as KBr (400–4000 cm^−1^) (Bruker, Billerica, MA, USA). Ultraviolet–visible spectroscopy (UV-Vis) absorption and fluorescence spectra were recorded on a Shimadzu UV-2550 spectrometer (Shimadzu, Tokyo, Japan) and F-7000 FL spectrometer (Hitachi, Tokyo, Japan), respectively. X-ray single crystal structure determinations were carried out on a Bruker APEX-II CCD diffractometer (Bruker AVANCE, Billerica, MA, USA). Supplementary crystallographic data for this paper have been deposited at the Cambridge Crystallographic Data Centre (1562395 and 1562396 for the coordination compounds **1** and **2**) and can be obtained free of charge via www.ccdc.cam.ac.uk/conts/retrieving.html.

### 3.2. Preparation of Ligand H_3_L

**Scheme 1:**
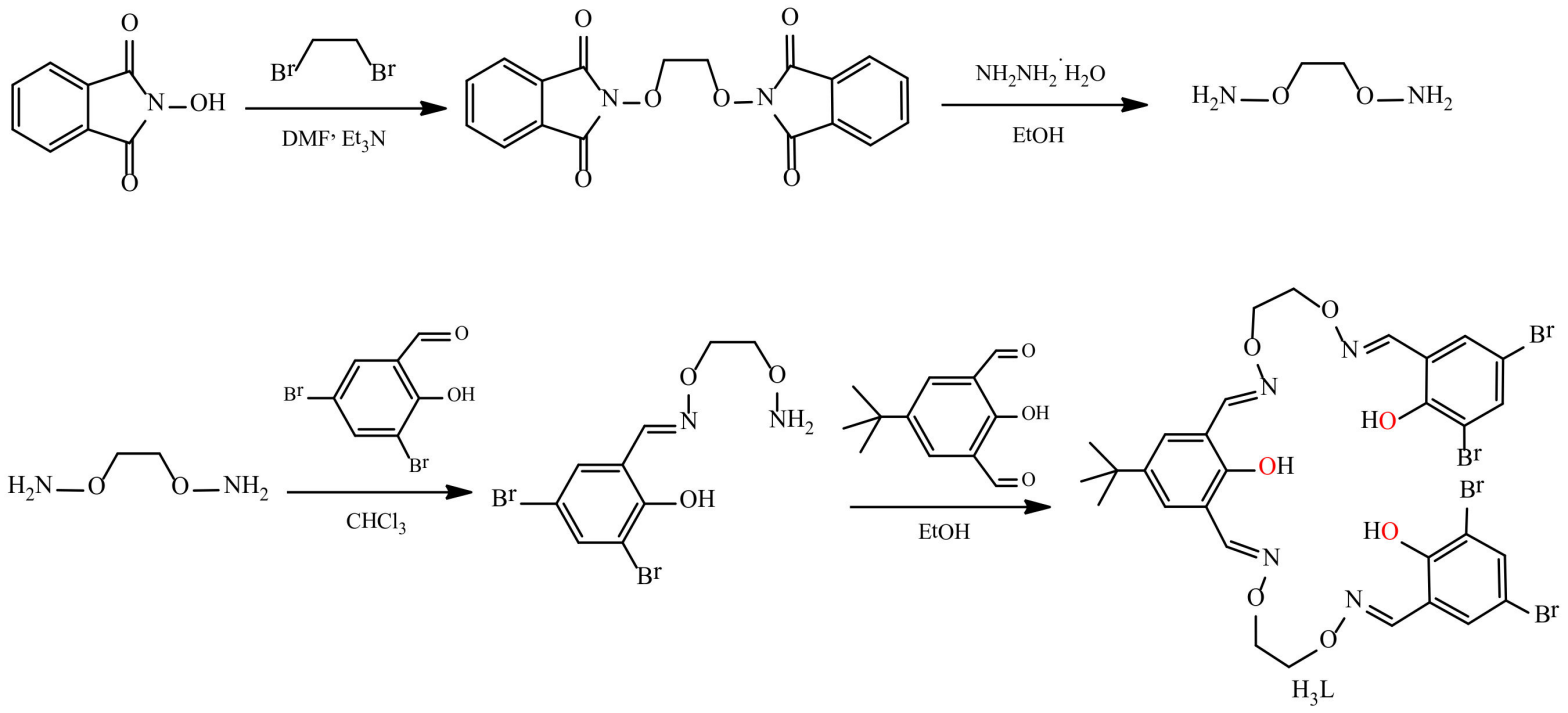
Synthetic route to the bis(salamo)-like tetraoxime ligand H_3_L.

1,2-Bis(aminooxy)ethane was synthesized according to an analogous method reported earlier [71]. Yield, 78.2%. Anal. Calcd. for C_2_H_8_N_2_O_2_: C, 26.08; H, 8.76; N, 30.42. Found: C, 25.38; H, 8.20; N, 29.76%. The synthetic route to novel bis(salamo)-like tetraoxime ligand (H_3_L) is shown in Scheme 1.

Next, the chloroform solution of 3,5-dibromosalicylaldehyde was added to 1,2-bis(aminooxy)ethane by drop to obtain a monooxime compound 2-[*O*-(1-ethyloxyamide)]oxime-4,6-dibromophenol. Last, the monooxime compound was reacted with 4-*tert*-butyl-2,6-diformylphenol (2:1) in the ethanol solvent after purification by the recrystallization method so as to obtain the symmetric bis(salamo)-like tetraoxime ligand H_3_L. Yield, 89.5%. m.p. 122–123 °C. Anal. Calcd. for C_30_H_30_N_4_O_7_Br_4_: C, 41.03; H, 3.44; N, 6.38. Found: C, 40.85; H, 3.32; N, 5.99%. ^1^H-NMR (400 MHz, DMSO) *δ* 10.37 (s, 1H), 10.04 (s, 1H), 8.45 (d, *J* = 1.8 Hz, 4H), 8.29 (s, 1H), 7.59 (s, 2H), 7.57 (d, *J* = 2.6 Hz, 2H), 7.51 (d, *J* = 2.6 Hz, 2H), 4.44 (s, 8H), 1.21 (s, 9H).

### 3.3. Syntheses of the Coordination Compounds **1** and **2**

The coordination compounds **1** and **2** were synthesized by the reaction of H_3_L with Co(OAc)_2_·4H_2_O and Zn(OAc)_2_·2H_2_O, respectively. A solution of Co(OAc)_2_·4H_2_O (4.98 mg, 0.02 mmol) in methanol (2 mL) was added dropwise to a solution of H_3_L (8.8 mg, 0.01 mmol) in dichloromethane (3 mL). The color of the mixing solution turned to bronzing immediately, and then continuous stirring for 0.5 h at room temperature. The mixture was filtered and the filtrate was allowed to stand at room temperature for about two weeks. The solvent was partially evaporated and obtained brown, block-shaped single crystals suitable for X-ray crystallographic analysis with a yield of 76.4%. Anal. Calcd. for [Co_2_(L)(OCH_3_)][Co_2_(L)(OAc)] (C_63_H_60_Br_8_Co_4_N_8_O_17_): C, 36.27; H, 2.76; N, 5.53; Co, 12.18. Found: C, 36.52; H, 2.64; N, 5.27; Co,11.85%.

The coordination compound **2** was prepared by the same method as that of the coordination compound **1**. A solution of Zn(OAc)_2_·2H_2_O (4.38 mg, 0.02 mmol) in methanol (2 mL) was added dropwise to a solution of H_3_L (8.8 mg, 0.01 mmol) in chloroform (3 mL). The color of the mixing solution turned to yellow immediately, and then continuous stirring for 0.5 h at room temperature. The mixture was filtered and the filtrate was allowed to stand at room temperature for about two weeks, the solvent was partially evaporated and obtained bright-yellow, block-shaped crystals. Yield, 71.6%. Anal. Calcd. for [Zn_2_(L)(OCH_3_)][Zn_2_(L)(OAc)] (C_63_H_60_Br_8_Zn_4_N_8_O_17_): C, 35.85; H, 2.79; N, 5.46; Zn, 12.58 %. Found: C, 36.18; H, 2.71; N, 5.32; Zn, 12.36%.

### 3.4. X-ray Structure Determination of the Coordination Compounds **1** and **2**

X-ray diffraction data were collected on a Bruker APEX-II CCD diffractometer (296(2) K) for the coordination compounds **1** and **2** using graphite monochromatized Mo-*K_α_* radiation (*λ* = 0.71073 Å). Unit cell parameters were determined by the least-squares analyses. The LP factor and Semi-empirical absorption corrections were applied to the intensity data. The structures were solved by the direct method (SHELXS-2016), and all hydrogen atoms were added theoretically. All non-hydrogen atoms were refined anisotropically using a full-matrix least-squares procedure on *F*^2^ with SHELXL-2016 (Bruker AVANCE, Billerica, MA, USA). Anisotropic thermal parameters were assigned to all non-hydrogen atoms. Contributions to scattering due to these highly disordered solvent molecules were removed using the SQUEEZE routine of PLATON, the structures were then refined again using the data generated. The hydrogen atoms were generated geometrically. Crystallographic data and refinement parameters for the coordination compounds **1** and **2** are given in Table 4.

## 4. Conclusions

We have designed and synthesized a novel symmetric bis(salamo)-like tetraoxime ligand H_3_L, and two unusual dinuclear coordination compounds **1** and **2**, [M_2_(L)(OCH_3_)][M_2_(L)(OAc)] (M^II^ = Co^II^ and Zn^II^). X-ray crystal structure analyses of the coordination compounds **1** and **2** reveal that the unit cell of the two coordination compounds contains two crystallographically and chemically independent dinuclear metal coordination compounds. The supramolecular structures of the coordination compounds **1** and **2** are different from each other, the coordination compound **1** forms a 3D supramolecular structure and the coordination compound **2** forms a 0D dimer structure by the inter-molecular hydrogen bond interactions. Furthermore, the fluorescence spectra of the coordination compounds **1** and **2** indicates that the coordination of Co^II^ and Zn^II^ ions leads to the fluorescence quenching and enhancing of H_3_L, respectively, which can be further studied as a new type of fluorescent material.

## Figures and Tables

**Table 1:** Selected bond lengths (Å) for the coordination compounds **1** and **2**.

Coordination Compound 1					
Bonds Lengths (Å)		Bonds Lengths (Å)		Bonds Lengths (Å)	
Co1-O1	1.952(3)	Co2-N3	2.060(3)	Co3-O16	2.138(3)
Co1-O8	1.961(3)	Co2-O4	2.069(3)	Co3-O17	2.198(3)
Co1-N2	2.033(3)	Co2-N4	2.083(4)	Co4-O15	1.917(3)
Co1-N1	2.045(3)	Co3-O12	2.021(3)	Co4-O9	2.022(3)
Co1-O4	2.071(3)	Co3-N5	2.047(4)	Co4-N7	2.027(3)
Co2-O7	1.954(3)	Co3-N6	2.063(3)	Co4-O12	2.086(3)
Co2-O8	1.961(3)	Co3-O9	2.074(3)	Co4-N8	2.139(3)
**Coordination Compound 2**					
**Bonds Lengths (Å)**		**Bonds Lengths (Å)**		**Bonds Lengths (Å)**	
Zn1-O17	1.954(5)	Zn2-O16	1.951(5)	Zn3-N6	2.115(5)
Zn1-O14	2.082(4)	Zn2-N3	2.103(6)	Zn3-N5	2.103(5)
Zn1-O15	1.978(4)	Zn2-N4	2.111(5)	Zn4-O4	2.096(4)
Zn1-N1	2.088(6)	Zn3-O4	2.047(4)	Zn4-O9	1.941(4)
Zn1-N2	2.101(5)	Zn3-O3	2.056(4)	Zn4-O3	2.027(4)
Zn2-O14	2.057(4)	Zn3-O7	2.205(5)	Zn4-N8	2.031(5)
Zn2-O15	1.991(5)	Zn3-O8	2.138(5)	Zn4-N7	2.180(5)

**Table 2:** Putative hydrogen bond interactions (Å, °) for the coordination compounds **1** and **2**.

Coordination Compound 1					
D‒X···A	d(D‒X)	d(X···A)	d(D···A)	∠D‒X···A	Symmetry Code
C9‒H9A···O13	0.97	2.44	3.231(6)	138	
C10‒H10···O15	0.93	2.43	3.130(5)	132	
C40‒H40A···Br8	0.97	2.90	3.776(6)	151	−*x*,1−*y*,1−*z*
C43‒H43···O7	0.93	2.60	3.477(6)	158	
C49‒H49A···O16	0.97	2.46	3.379(6)	159	
C8‒H8A···Br8	0.97	3.92	3.019(5)	154	
C61‒H61A···Br2	0.96	2.99	3.280(6)	99	
C36‒H36···Br7	0.93	3.81	3.044(5)	140	
C39‒H39B···Br6	0.97	2.96	3.895(5)	161	
**Coordination Compound 2**					
**D‒X···A**	**d(D‒X)**	**d(X···A)**	**d(D···A)**	**∠D‒X···A**	**Symmetry Code**
C6‒H6···O16	0.93	2.52	3.394(8)	157	
C11‒H11···O16	0.93	2.57	3.439(7)	156	
C22‒H22A···O8	0.97	2.43	3.374(8)	163	
C44‒H44···O9	0.93	2.40	3.070(9)	129	
C55‒H55···O10	0.93	2.59	3.297(9)	133	[1−x,1−y,1−z]

**Table 3:** Main IR bands (cm^−1^) for the ligand H_3_L and its coordination compounds **1** and **2**.

Coordination Compound	*ν*_(C=N)_	*ν*_(Ar–O)_	*ν*_(Co–O)_	*ν*_(Co–N)_
H_3_L	1611	1265		
**1**	1619	1258	447	512
**2**	1621	1261	453	519

**Table 4:** Crystallographic data and refinement parameters for the coordination compounds **1** and **2**.

Coordination Compound	1	2
Empirical formula	C_63_H_60_Br_8_Co_4_N_8_O_17_	C_63_H_60_Br_8_Zn_4_N_8_O_17_
Molecular weight	2076.19	2101.95
Color	Brown	Bright-yellow
Crystal size, mm	0.22 × 0.18 × 0.16	0.22 × 0.18 × 0.17
Habit	Block-shaped	Block-shaped
Crystal system	Triclinic	Triclinic
Space group	*P*-1	*P*-1
Unit cell dimension		
*a*, Å	15.244(2)	13.4501(6)
*b*, Å	18.674(3)	18.6963(9)
*c*, Å	21.356(3)	19.6467(8)
*α*, °	109.512(4)	72.7450(10)
*β*, °	97.335(4)	72.5280(10)
*γ*, °	109.429(4)	88.801(2)
Volume, Å^3^	5205.4(14)	4487.9(3)
*Z*	2	2
Calculated density, mg·m^−3^	1.325	1.555
Absorption coefficient, mm^−1^	3.747	4.675
*F* (000)	2036	2060
*θ* range for data collection, °	1.050 to 27.000	2.224 to 25.010
*h*/*k*/*l* (min, max)	−19, 16/−22, 23/−27, 26	−11, 15/−22, 21/−23, 23
Reflections collected	37,783	32,780
Completeness	96.6%	99.4%
Data/restraints/parameters	22,025/1/909	15,711/0/909
Final *R* indices [*I >* 2*σ*(*I*)]	*R*_1_ = 0.0438, *wR*_2_ = 0.0932	*R*_1_ = 0.0523, *wR*_2_ = 0.1353
*R* indices (all data)	*R*_1_ = 0.0762, *wR*_2_ = 0.0977	*R*_1_ = 0.0888, *wR*_2_ = 0.1557
Largest diff. peak and hole (e·Å^−3^)	1.677, −0.861	1.434, −0.914

## Supplementary Materials

The following are available online at http://www.mdpi.com/1420-3049/23/5/1141/s1.
