# Supplementary material for: Unprecedented Fluorescent Dinuclear CoII and ZnII Coordination Compounds with a Symmetric Bis(salamo)-Like Tetraoxime
Source: Molecules. 2018 May 10;23(5):1141. doi: 10.3390/molecules23051141 (PMC6099918; doi:10.3390/molecules23051141)

checkCIF/PLATON report

You have not supplied any structure factors. As a result the full set of tests cannot be run.

THIS REPORT IS FOR GUIDANCE ONLY. IF USED AS PART OF A REVIEW PROCEDURE FOR PUBLICATION, IT SHOULD NOT REPLACE THE EXPERTISE OF AN EXPERIENCED CRYSTALLOGRAPHIC REFEREE.

No syntax errors found. CIF dictionary Interpreting this report

Datablock: 20171225lzw122_0m_a_sq

Bond precision: C-C = 0.0105 A Wavelength=0.71073

Cell:	a=13.4501(6)	b=18.6963(9)	c=19.6467(8)
	alpha=72.745(1)	beta=72.528(1)	gamma=88.801(2)
Temperature:	296 K		

	Calculated	Reported
Volume	4487.9(3)	4487.9(3)
Space group	P -1	P -1
Hall group	-P 1	-P 1
Moiety formula	C32 H30 Br4 N4 O9 Zn2, C31 H30 Br4 N4 O8 Zn2 [+ solvent]	C32 H30 Br4 N4 O9 Zn2, C31 H30 Br4 N4 O8 Zn2
Sum formula	C63 H60 Br8 N8 O17 Zn4 [+ solvent]	C63 H60 Br8 N8 O17 Zn4
Mr	2101.95	2101.95
Dx,g cm-3	1.556	1.555
Z	2	2
Mu (mm-1)	4.675	4.675
F000	2060.0	2060.0
F000’	2058.70	
h,k,lmax	15,22,23	15,22,23
Nref	15809	15711
Tmin,Tmax	0.379,0.452	
Tmin’	0.344	

Correction method= Not given

Data completeness= 0.994 Theta(max)= 25.010

R(reflections)= 0.0523(10738) wR2(reflections)= 0.1557(15711)

S = 1.049 Npar= 909

The following ALERTS were generated. Each ALERT has the format
test-name_ALERT_alert-type_alert-level.
Click on the hyperlinks for more details of the test.

Alert level C

```
PLAT052_ALERT_1_C Info on Absorption Correction Method   Not Given     Please Do !
PLAT057_ALERT_3_C Correction for Absorption Required   RT(exp) ...       1.19 Do !
PLAT221_ALERT_2_C Solv./Anion  Resd 2  C   Ueq(max)/Ueq(min) Range        5.7 Ratio
PLAT223_ALERT_4_C Solv./Anion  Resd 2  H   Ueq(max)/Ueq(min) Range        6.4 Ratio
PLAT230_ALERT_2_C Hirshfeld Test Diff for   O15      --C63       .        5.1 s.u.
PLAT242_ALERT_2_C Low    'MainMol' Ueq as Compared to Neighbors of        O15 Check
PLAT242_ALERT_2_C Low    'MainMol' Ueq as Compared to Neighbors of        C36 Check
PLAT341_ALERT_3_C Low Bond Precision on  C-C Bonds ...............    0.01046 Ang.
PLAT431_ALERT_2_C Short Inter HL..A Contact  Br1      ..O13              3.25 Ang.
```

Alert level G

```
PLAT395_ALERT_2_G Deviating  X-O-Y    Angle From 120 for O1             110.9 Degree
PLAT395_ALERT_2_G Deviating  X-O-Y    Angle From 120 for O2             110.7 Degree
PLAT395_ALERT_2_G Deviating  X-O-Y    Angle From 120 for O5             110.4 Degree
PLAT395_ALERT_2_G Deviating  X-O-Y    Angle From 120 for O6             111.5 Degree
PLAT395_ALERT_2_G Deviating  X-O-Y    Angle From 120 for O10            109.7 Degree
PLAT395_ALERT_2_G Deviating  X-O-Y    Angle From 120 for O11            109.5 Degree
PLAT395_ALERT_2_G Deviating  X-O-Y    Angle From 120 for O12            112.0 Degree
PLAT395_ALERT_2_G Deviating  X-O-Y    Angle From 120 for O13            110.0 Degree
PLAT432_ALERT_2_G Short Inter X...Y Contact  Br4      ..C44              3.32 Ang.
PLAT432_ALERT_2_G Short Inter X...Y Contact  O1       ..C31              2.98 Ang.
PLAT606_ALERT_4_G VERY LARGE Solvent Accessible VOID(S) in Structure        ! Info
PLAT869_ALERT_4_G ALERTS Related to the Use of SQUEEZE Suppressed           ! Info
PLAT933_ALERT_2_G Number of OMIT Records in Embedded .res File ...          4 Note
```

---

0 **ALERT level A** = Most likely a serious problem - resolve or explain
0 **ALERT level B** = A potentially serious problem, consider carefully
9 **ALERT level C** = Check. Ensure it is not caused by an omission or oversight
13 **ALERT level G** = General information/check it is not something unexpected

1 ALERT type 1 CIF construction/syntax error, inconsistent or missing data
16 ALERT type 2 Indicator that the structure model may be wrong or deficient
2 ALERT type 3 Indicator that the structure quality may be low
3 ALERT type 4 Improvement, methodology, query or suggestion
0 ALERT type 5 Informative message, check

It is advisable to attempt to resolve as many as possible of the alerts in all categories. Often the minor alerts point to easily fixed oversights, errors and omissions in your CIF or refinement strategy, so attention to these fine details can be worthwhile. In order to resolve some of the more serious problems it may be necessary to carry out additional measurements or structure refinements. However, the purpose of your study may justify the reported deviations and the more serious of these should normally be commented upon in the discussion or experimental section of a paper or in the "special_details" fields of the CIF. checkCIF was carefully designed to identify outliers and unusual parameters, but every test has its limitations and alerts that are not important in a particular case may appear. Conversely, the absence of alerts does not guarantee there are no aspects of the results needing attention. It is up to the individual to critically assess their own results and, if necessary, seek expert advice.

Publication of your CIF in IUCr journals

A basic structural check has been run on your CIF. These basic checks will be run on all CIFs submitted for publication in IUCr journals (*Acta Crystallographica*, *Journal of Applied Crystallography*, *Journal of Synchrotron Radiation*); however, if you intend to submit to *Acta Crystallographica Section C* or *E* or *IUCrData*, you should make sure that full publication checks are run on the final version of your CIF prior to submission.

Publication of your CIF in other journals

Please refer to the *Notes for Authors* of the relevant journal for any special instructions relating to CIF submission.

PLATON version of 30/01/2018; check.def file version of 30/01/2018

Datablock 20171225lzw122_0m_a_sq - ellipsoid plot

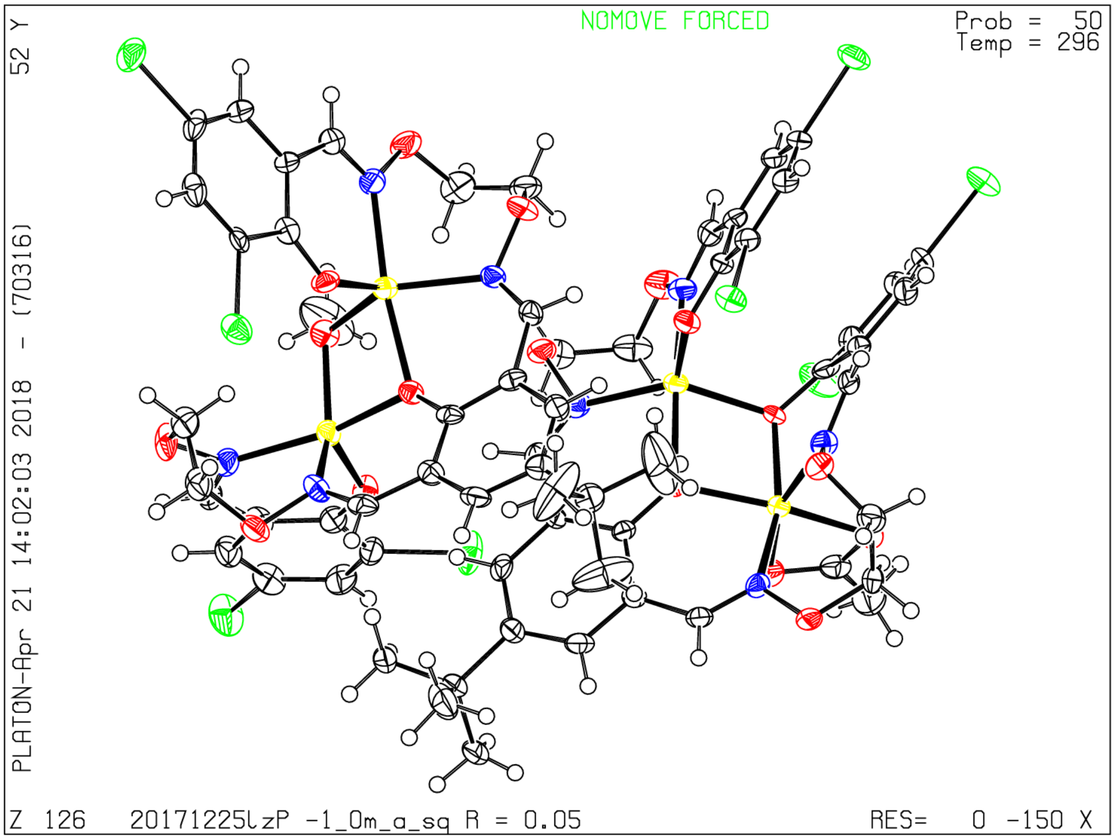

Supplement: Supplementary file 1 [file molecules-23-01141-s001.zip › Zn coordination compound checkcif report.pdf]
