# Supplementary material for: Unprecedented Fluorescent Dinuclear CoII and ZnII Coordination Compounds with a Symmetric Bis(salamo)-Like Tetraoxime
Source: Molecules. 2018 May 10;23(5):1141. doi: 10.3390/molecules23051141 (PMC6099918; doi:10.3390/molecules23051141)

checkCIF/PLATON report

You have not supplied any structure factors. As a result the full set of tests cannot be run.

THIS REPORT IS FOR GUIDANCE ONLY. IF USED AS PART OF A REVIEW PROCEDURE FOR PUBLICATION, IT SHOULD NOT REPLACE THE EXPERTISE OF AN EXPERIENCED CRYSTALLOGRAPHIC REFEREE.

No syntax errors found. CIF dictionary Interpreting this report

Datablock: x_sq

Bond precision: C-C = 0.0066 A Wavelength=0.71073

Cell:	a=15.244(2)	b=18.674(3)	c=21.356(3)
	alpha=109.512(4)	beta=97.335(4)	gamma=109.429(4)
Temperature:	291 K		

	Calculated	Reported
Volume	5205.3(13)	5205.4(14)
Space group	P -1	P -1
Hall group	-P 1	-P 1
Moiety formula	C32 H30 Br4 Co2 N4 O9, C31 H30 Br4 Co2 N4 O8 [+ solvent]	C32 H30 Br4 Co2 N4 O9, C31 H30 Br4 Co2 N4 O8
Sum formula	C63 H60 Br8 Co4 N8 O17 [+ solvent]	C63 H60 Br8 Co4 N8 O17
Mr	2076.11	2076.19
Dx,g cm-3	1.325	1.325
Z	2	2
Mu (mm-1)	3.747	3.747
F000	2036.0	2036.0
F000’	2035.17	
h,k,lmax	19,23,27	19,23,27
Nref	22753	22025
Tmin,Tmax	0.450,0.549	0.490,0.590
Tmin’	0.434	

Correction method= # Reported T Limits: Tmin=0.490 Tmax=0.590
AbsCorr = MULTI-SCAN

Data completeness= 0.968 Theta(max)= 27.000

R(reflections)= 0.0438(13952) wR2(reflections)= 0.0977(22025)

S = 1.005 Npar= 909

The following ALERTS were generated. Each ALERT has the format
test-name_ALERT_alert-type_alert-level.
Click on the hyperlinks for more details of the test.

Alert level C

```
PLAT230_ALERT_2_C Hirshfeld Test Diff for   O9       --C57       .        6.7 s.u.
PLAT230_ALERT_2_C Hirshfeld Test Diff for   O14      --C39       .        6.0 s.u.
PLAT230_ALERT_2_C Hirshfeld Test Diff for   C43      --C44       .        5.7 s.u.
PLAT230_ALERT_2_C Hirshfeld Test Diff for   Br3      --C23       .        6.0 s.u.
PLAT230_ALERT_2_C Hirshfeld Test Diff for   C3       --C4        .        5.2 s.u.
PLAT341_ALERT_3_C Low Bond Precision on  C-C Bonds ...............    0.00665 Ang.
PLAT412_ALERT_2_C Short Intra XH3 .. XHn     H14      ..H30B             1.82 Ang.
```

Alert level G

```
PLAT003_ALERT_2_G Number of Uiso or Uij Restrained non-H Atoms ...          2 Report
PLAT154_ALERT_1_G The s.u.'s on the Cell Angles are Equal ..(Note)      0.004 Degree
PLAT177_ALERT_4_G The CIF-Embedded .res File Contains DELU Records          1 Report
PLAT232_ALERT_2_G Hirshfeld Test Diff (M-X) Co3      --O17       .        7.8 s.u.
PLAT335_ALERT_2_G Check Large C6 Ring C-C Range C42      -C47            0.16 Ang.
PLAT395_ALERT_2_G Deviating  X-O-Y    Angle From 120 for O10            108.4 Degree
PLAT395_ALERT_2_G Deviating  X-O-Y    Angle From 120 for O11            112.1 Degree
PLAT395_ALERT_2_G Deviating  X-O-Y    Angle From 120 for O13            110.2 Degree
PLAT395_ALERT_2_G Deviating  X-O-Y    Angle From 120 for O14            110.5 Degree
PLAT395_ALERT_2_G Deviating  X-O-Y    Angle From 120 for O2             108.6 Degree
PLAT395_ALERT_2_G Deviating  X-O-Y    Angle From 120 for O3             109.1 Degree
PLAT395_ALERT_2_G Deviating  X-O-Y    Angle From 120 for O5             110.6 Degree
PLAT395_ALERT_2_G Deviating  X-O-Y    Angle From 120 for O6             110.4 Degree
PLAT432_ALERT_2_G Short Inter X...Y Contact  Br2      ..C61              3.28 Ang.
PLAT432_ALERT_2_G Short Inter X...Y Contact  O11      ..C62              2.95 Ang.
PLAT434_ALERT_2_G Short Inter HL..HL Contact Br3      ..Br6              3.60 Ang.
PLAT606_ALERT_4_G VERY LARGE Solvent Accessible VOID(S) in Structure        ! Info
PLAT794_ALERT_5_G Tentative Bond Valency for Co1       (II)      .       2.04 Info
PLAT794_ALERT_5_G Tentative Bond Valency for Co2       (II)      .       1.98 Info
PLAT794_ALERT_5_G Tentative Bond Valency for Co3       (II)      .       1.99 Info
PLAT794_ALERT_5_G Tentative Bond Valency for Co4       (II)      .       1.93 Info
PLAT802_ALERT_4_G CIF Input Record(s) with more than 80 Characters          1 Info
PLAT860_ALERT_3_G Number of Least-Squares Restraints .............          1 Note
PLAT869_ALERT_4_G ALERTS Related to the Use of SQUEEZE Suppressed           ! Info
PLAT933_ALERT_2_G Number of OMIT Records in Embedded .res File ...          6 Note
```

0 **ALERT level A** = Most likely a serious problem - resolve or explain
0 **ALERT level B** = A potentially serious problem, consider carefully
7 **ALERT level C** = Check. Ensure it is not caused by an omission or oversight
25 **ALERT level G** = General information/check it is not something unexpected

1 ALERT type 1 CIF construction/syntax error, inconsistent or missing data
21 ALERT type 2 Indicator that the structure model may be wrong or deficient
2 ALERT type 3 Indicator that the structure quality may be low
4 ALERT type 4 Improvement, methodology, query or suggestion
4 ALERT type 5 Informative message, check

It is advisable to attempt to resolve as many as possible of the alerts in all categories. Often the minor alerts point to easily fixed oversights, errors and omissions in your CIF or refinement strategy, so attention to these fine details can be worthwhile. In order to resolve some of the more serious problems it may be necessary to carry out additional measurements or structure refinements. However, the purpose of your study may justify the reported deviations and the more serious of these should normally be commented upon in the discussion or experimental section of a paper or in the "special_details" fields of the CIF. checkCIF was carefully designed to identify outliers and unusual parameters, but every test has its limitations and alerts that are not important in a particular case may appear. Conversely, the absence of alerts does not guarantee there are no aspects of the results needing attention. It is up to the individual to critically assess their own results and, if necessary, seek expert advice.

Publication of your CIF in IUCr journals

A basic structural check has been run on your CIF. These basic checks will be run on all CIFs submitted for publication in IUCr journals (*Acta Crystallographica*, *Journal of Applied Crystallography*, *Journal of Synchrotron Radiation*); however, if you intend to submit to *Acta Crystallographica Section C* or *E* or *IUCrData*, you should make sure that full publication checks are run on the final version of your CIF prior to submission.

Publication of your CIF in other journals

Please refer to the *Notes for Authors* of the relevant journal for any special instructions relating to CIF submission.

PLATON version of 23/04/2018; check.def file version of 23/04/2018

Datablock x_sq - ellipsoid plot

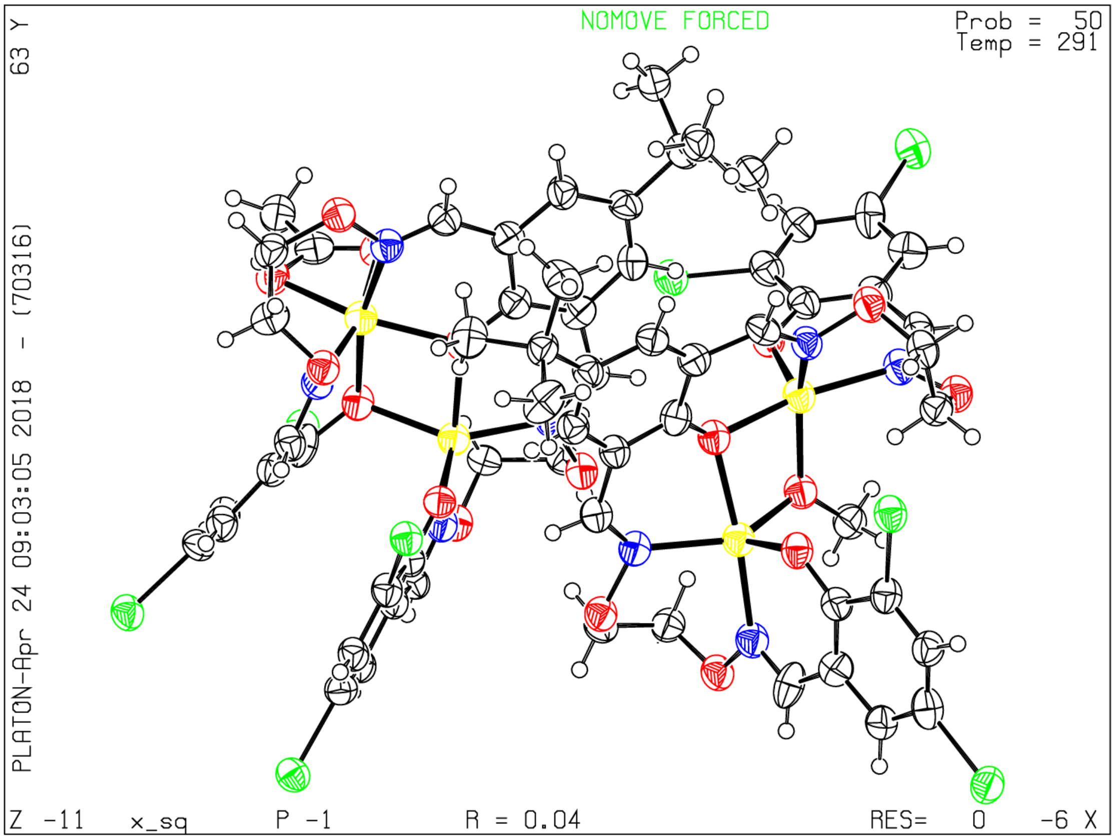

Supplement: Supplementary file 1 [file molecules-23-01141-s001.zip › Co coordination compound checkcif report.pdf]
