# Supplementary material for: Unprecedented Fluorescent Dinuclear CoII and ZnII Coordination Compounds with a Symmetric Bis(salamo)-Like Tetraoxime
Source: Molecules. 2018 May 10;23(5):1141. doi: 10.3390/molecules23051141 (PMC6099918; doi:10.3390/molecules23051141)
Supplement: Supplementary file 1 [file molecules-23-01141-s001.zip › Supplementary Information.doc]

**Table S1.** Selected bond angles (°) for the coordination compounds **1** and **2**.

| Coordination compound **1** | | | | | | | | | |
| --- | --- | --- | --- | --- | --- | --- | --- | --- | --- |
| Angles (**°**) |  | | Angles (**°**) |  | | Angles (**°**) | |  | |
| O1-Co1-O8 | 101.51(11) | O8-Co2-N4 | | 99.92(13) | | O16-Co3-O17 | | 62.27(11) | |
| O1-Co1-N2 | 119.82(13) | N3-Co2-N4 | | 89.06(13) | | O12-Co3-C62 | | 118.93(14) | |
| O8-Co1-N2 | 135.01(13) | O4-Co2-N4 | | 162.54(13) | | N5-Co3-C62 | | 133.65(15) | |
| O1-Co1-N1 | 91.36(13) | O12-Co3-N5 | | 104.76(12) | | N6-Co3-C62 | | 103.70(13) | |
| O8-Co1-N1 | 102.61(13) | O12-Co3-N6 | | 86.01(12) | | O9-Co3-C62 | | 87.89(12) | |
| N2-Co1-N1 | 93.90(14) | N5-Co3-N6 | | 93.49(13) | | O16-Co3-C62 | | 32.08(13) | |
| O1-Co1-O4 | 96.38(11) | O12-Co3-O9 | | 80.34(10) | | O17-Co3-C62 | | 30.35(13) | |
| O8-Co1-O4 | 77.55(11) | N5-Co3-O9 | | 84.69(12) | | O15-Co4-O9 | | 102.52(12) | |
| N2-Co1-O4 | 80.79(12) | N6-Co3-O9 | | 165.25(12) | | O15-Co4-N7 | | 119.21(12) | |
| N1-Co1-O4 | 172.08(12) | O12-Co3-O16 | | 150.11(11) | | O9-Co4-N7 | | 138.01(13) | |
| O7-Co2-O8 | 106.55(11) | N5-Co3-O16 | | 101.99(12) | | O15-Co4-O12 | | 104.13(11) | |
| O7-Co2-N3 | 114.64(12) | N6-Co3-O16 | | 105.51(12) | | O9-Co4-O12 | | 80.02(10) | |
| O8-Co2-N3 | 138.09(13) | O9-Co3-O16 | | 89.17(10) | | N7-Co4-O12 | | 85.22(12) | |
| O7-Co2-O4 | 109.12(11) | O12-Co3-O17 | | 89.85(11) | | O15-Co4-N8 | | 87.56(13) | |
| O8-Co2-O4 | 77.62(11) | N5-Co3-O17 | | 163.86(12) | | O9-Co4-N8 | | 94.18(12) | |
| N3-Co2-O4 | 81.91(12) | N6-Co3-O17 | | 94.34(12) | | N7-Co4-N8 | | 92.19(13) | |
| O7-Co2-N4 | 88.21(13) | O9-Co3-O17 | | 91.20(11) | | O12-Co4-N8 | | 167.80(12) | |
| Coordination compound **2** | | | | | | | | | |
| Angles (**°**) |  | Angles (**°**) | | |  | | Angles (**°**) | |  |
| O17-Zn1-O14 | 94.96(18) | O16-Zn2-O14 | | | 108.56(18) | | N6-Zn3-O7 | | 160.0(2) |
| O17-Zn1-N1 | 103.66(19) | O16-Zn2-O15 | | | 110.7(2) | | N6-Zn3-O8 | | 99.57(19) |
| O17-Zn1-O15 | 90.0(2) | O16-Zn2-N3 | | | 111.6(2) | | N5-Zn3-O7 | | 96.12(19) |
| O17-Zn1-N2 | 118.2(2) | O16-Zn2-N4 | | | 89.3(2) | | N5-Zn3-O8 | | 108.37(19) |
| O14-Zn1-N1 | 172.66(19) | N3-Zn2-N4 | | | 89.5(2) | | N5-Zn3-N6 | | 91.0(2) |
| O14-Zn1-N2 | 81.15(18) | O4-Zn3-O3 | | | 79.26(16) | | O4-Zn4-N7 | | 166.4(2) |
| O15-Zn1-O14 | 79.20(17) | O4-Zn3-O7 | | | 94.58(17) | | O9-Zn4-O4 | | 106.35(18) |
| O15-Zn1-N1 | 104.9(2) | O4-Zn3-O8 | | | 151.86(17) | | O9-Zn4-O3 | | 103.90(18) |
| O15-Zn1-N2 | 134.9(2) | O4-Zn3-N6 | | | 104.58(19) | | O9-Zn4-N8 | | 118.4(2) |
| N1-Zn1-N2 | 91.7(2) | O4-Zn3-N5 | | | 85.62(18) | | O9-Zn4-N7 | | 86.7(2) |
| O14-Zn2-N3 | 80.52(18) | O3-Zn3-O7 | | | 93.98(18) | | O3-Zn4-O4 | | 78.78(16) |
| O14-Zn2-N4 | 161.8(2) | O3-Zn3-O8 | | | 89.12(18) | | O3-Zn4-N8 | | 137.4(2) |
| O15-Zn2-O14 | 79.49(17) | O3-Zn3-N6 | | | 84.23(18) | | O3-Zn4-N7 | | 94.45(19) |
| O15-Zn2-N3 | 137.1(2) | O3-Zn3-N5 | | | 162.43(19) | | N8-Zn4-O4 | | 84.36(18) |
| O15-Zn2-N4 | 98.2(2) | O8-Zn3-O7 | | | 60.47(17) | | N8-Zn4-N7 | | 93.0(2) |

^1^H NMR spectra of ligand H_3_L
